# Seasonal shifts in the competitive ability of macroalgae influence the outcomes of coral–algal competition

**DOI:** 10.1098/rsos.201797

**Published:** 2020-12-23

**Authors:** Kristen T. Brown, Dorothea Bender-Champ, Ove Hoegh-Guldberg, Sophie Dove

**Affiliations:** 1 School of Biological Sciences, The University of Queensland, St Lucia, Queensland 4072, Australia; 2 Australian Research Council Centre of Excellence for Coral Reef Studies, The University of Queensland, St Lucia, Queensland 4072, Australia; 3 Global Change Institute, The University of Queensland, St Lucia, Queensland 4072, Australia

**Keywords:** coral–algal interaction, competition, macroalgae, seasonality, coral reefs

## Abstract

Understanding the effects of natural processes on coral–algal competition is an important step in identifying the role of macroalgae in perturbed coral reef ecosystems. However, studies investigating coral–algal interactions are often conducted in response to a disturbance, and rarely incorporate seasonal variability. Here, naturally occurring coral–algal interactions were assessed *in situ* four times a year over 2 years across eight sites spanning diverse benthic communities. In over 6500 recorded coral–algal interactions, cyanobacteria and turf algae were found to be the most damaging regardless of season, resulting in visible damage to coral in greater than 95% of interactions. Macroalgae that primarily compete using chemical mechanisms were found to be more damaging than those that compete using physical mechanisms (e.g. abrasion), with both groups demonstrating decreased competitive ability in summer. While crustose coralline algae were the least damaging to competing coral, during summer, it became three times more competitive. Our results demonstrate that the competitive ability of macroalgae and the outcomes of coral–algal competition can fluctuate in seasonal cycles that may be related to biomass, production of chemical defences and/or physical toughness. The results of this study have important implications for understanding the trajectory and resilience of coral reef ecosystems into the future.

## 1. Background

Macroalgae from coral reef ecosystems vary in space and time owing to a combination of biotic (e.g. herbivory) and abiotic (e.g. temperature, wave action) processes [1–3]. Anthropogenic activities, such as land-clearing and fertilization for agriculture, the loss of herbivores because of overexploitation or thermal stress, can disrupt the natural balance leading to reductions in reef-building coral and increases in macroalgal cover [4,5]. An increase in the abundance of macroalgae can lead to increases in coral–algal competition [6,7], which may play an important role in the degradation of coral reefs [8]. Given rapidly shifting coral reef ecosystem dynamics [9], macroalgae and their interactions with corals are more relevant than ever.

On the Great Barrier Reef, macroalgae display latitudinal, regional, within reef and seasonal patterns. Generally, macroalgal abundance increases latitudinally from north to south, longitudinally from offshore to inshore, within a reef system from the reef slope to reef flat and in the austral winter and spring [2–4,10,11]. In reef systems that are relatively unaffected by anthropogenic disturbances (e.g. eutrophication, overfishing), seasonal shifts in water motion, temperature, light and nutrients are the principal drivers of algal abundance [1,2,12,13]. While seasonality has been found to influence the frequency of coral–algal contact [2], it is not known if seasonal shifts in algal abundance relate to the competitive ability of macroalgae.

**Figure 1.**
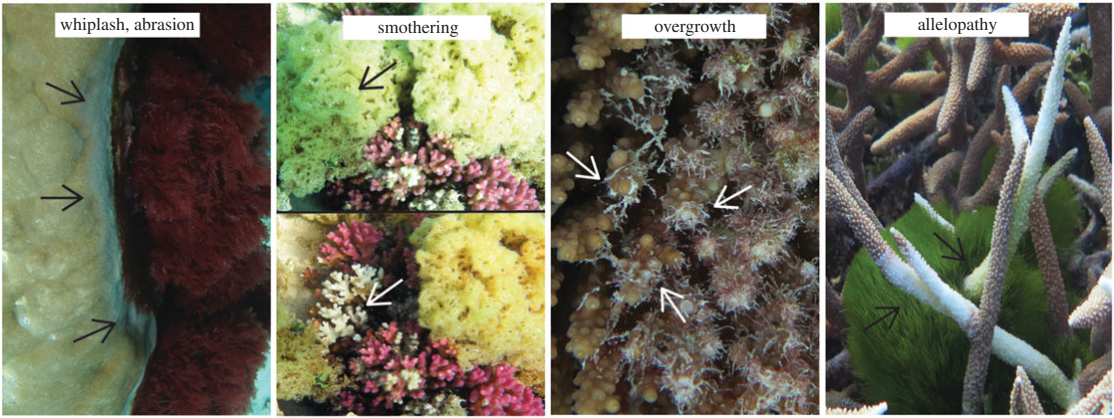
Common examples of coral–algal interactions at Heron Island, southern Great Barrier Reef. All interactions represent the outcome coral losing. From left to right: whiplash or abrasion between massive *Porites* and *Plocamium*; an ephemeral bloom of *Hydroclathrus* smothering *Pocillopora* (where the top image is prior to macroalgal removal and bottom after); turf algae actively overgrowing and killing live tabulate *Acropora* tissue; and the allelopathic alga *Chlorodesmis fastigiata* resulting in damage to staghorn *Acropora* that extends past the area of direct contact. Arrows highlight the interaction zone.

Competition between coral and macroalgae may take either of two forms: direct aggressive behaviour or indirect defensive behaviour [14]. Several prominent competitive mechanisms, reviewed by McCook *et al*. [14] and Chadwick & Morrow [15], include pre-emption, overgrowth, smothering, abrasion, shading, allelopathy and microbial enhancement (figure 1). Studies mostly demonstrate negative impacts of direct contact by macroalgae, resulting in reductions to coral growth, calcification, fecundity and survivorship [16–19]. Macroalgal growth, pigmentation and chemical defences can also be compromised by contact with corals [20,21]. A number of studies have determined that the outcomes of coral–algal interactions are dependent on a range of factors, including the kind of coral and algae involved, the size and growth form of the coral colony and the proportion of macroalgae in contact with the coral [2,22–26]. Investigations into whether seasonal changes can alter the competitive ability of macroalgae and the outcomes of coral–algal competition have been less clear [6].

Our study investigates whether the outcomes of coral–algal competition are influenced by seasonal changes in environmental conditions on the reef system Heron Reef (23.442° S, 151.914° E), on the southern Great Barrier Reef. Heron Island is a small (800 × 300 m) offshore coral cay with a large platform reef environment that is also home to a tourist resort and scientific research station (University of Queensland). Owing to being 50 km offshore and being protected by the Great Barrier Reef Marine Park Authority since 1975, impacts from eutrophication and sedimentation owing to agriculture are non-existent, as is the overharvesting of herbivores [27]. Heron Reef was among the least affected reefs from the cumulative footprint of 2016–2017 coral bleaching events on the Great Barrier Reef [28]. Therefore, macroalgal dynamics were principally driven by pronounced seasonal oscillations in environmental conditions such as temperature and light [2].

We assessed naturally occurring coral–algal interactions *in situ* four times a year over 2 years (2015–2016) across eight sites spanning the geomorphological habitats of Heron Reef. By examining a range of habitats across seasons, we are able to catalogue a comprehensive set of interactions that include diverse coral and macroalgal communities [2,29] (table 1). With this information, we investigated which macroalgal groups or types are the most damaging to competing coral, and within these groups, if the competitive ability of macroalgae varies with season. The results of this study provide a better understanding of the mechanisms by which coral and macroalgae compete and the seasons in which macroalgae are the most competitive.

**Table 1.** Groups of macroalgae. (Within each group or taxa, genera indicate recorded taxa, where ‘e.g.’ refers to commonly observed taxa from Heron Reef. Chemical macroalgae compete through the transfer of allelochemicals via direct contact, whereas physical macroalgae compete primarily using physical mechanisms (e.g. abrasion).)

group or taxa	growth form or genera	references
articulate coralline algae (ACA)	e.g. *Amphiroa*	
chemical	*Amansia, Asparagopsis, Chlorodesmis, Dictyota, Laurencia, Lobophora, Padina, Plocamium*	[30–32]
crustose coralline algae (CCA)	e.g. reef crest (*Porolithon, Lithophyllum*), reef slope (*Lithothamnion, Mesophyllum*)	[33]
cyanobacteria	e.g. *Lyngbya*	
*Halimeda*	e.g. reef flat *(H*. *discoidea*, *H. macroloba),* * *reef slope *(H. heteromorpha, H. macrophysa)*	[34,35]
physical	*Avrainvillea, Caulerpa, Chnoospora, Codium, Colpomenia, Dictyosphaeria, Hydroclathrus, Hypnea, Sargassum, Turbinaria, Valonia*	[16,36,37]
turf algae	e.g. *Feldmannia*	[26]

## 2. Methods

A total of eight surveys, representing each austral season twice, were conducted during the 2 year period between January 2015 and November 2016. Surveys were performed across the same eight sites during each visit to incorporate the breadth of coral and macroalgal communities that naturally occur across the geomorphological habitats of Heron Reef [2,29] (electronic supplementary material, figure S1). Coral–algal interactions were recorded using a modified line intercept approach first described by Barott *et al*. [38] and subsequently modified by Brown *et al*. [2]. At each site, 3 × 15 m transects were established coursing north, east and west from a permanent reference point. Within a 1 m belt, every coral colony was examined and any colony that was physically touching algae identified and recorded (figure 1). A single coral colony could be involved in multiple competitive interactions with different macroalgal taxa or groups [24]. The types of interacting corals and macroalgae were recorded to genus level, with the exception of cyanobacteria, turf algae, articulate and crustose coralline algae (CCA), which generally cannot be identified to genus level *in situ* [39]. The outcome of each interaction was recorded, with the three outcomes being: 1, coral overgrowing algae (coral ‘winning’); 2, algae overgrowing coral resulting in discoloration to the coral (coral ‘losing’); and 3, seemingly neutral (figure 1; see also fig. 2 in [22] and fig. 3 in [25]) [22,24,25]. Pigmentation loss at the interaction zone was clearly distinguishable from any other irregularities found on coral colonies (figure 1). Where macroalgae were covering coral, it was removed to determine the outcome in the area underneath, as not all contact by macroalgae results in discoloration to the competing coral [19,25,36]. Individual colonies were not tracked through time; therefore, the long-term fate (i.e. beyond one season) was not investigated in this study. Seasonal means for benthic cover, temperature and irradiance for the study period are reported in Brown *et al*. [2].

**Figure 2.**
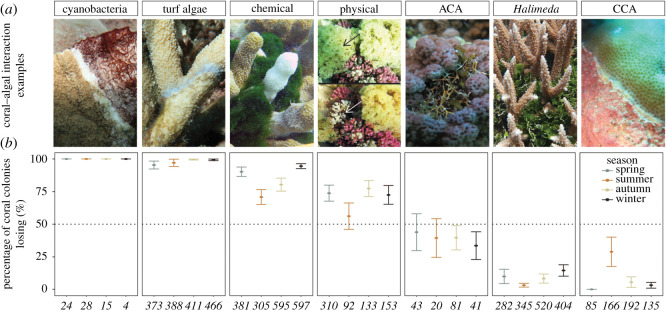
Coral–algal interaction outcomes by macroalgae type and season. Macroalgal group or taxa are indicated at the top of the figure. (*a*) Common examples of coral–algal interactions for each macroalgal group or taxa at Heron Island, southern Great Barrier Reef. For the physical interaction, two images indicate before (top) and after (bottom) macroalgal removal, with arrows highlighting the interaction zone. (*b*) Percentage of coral colonies (% ± s.e.) where corals are losing by macroalgal group or taxa. Italicized numbers indicate the total number of coral colonies evaluated per macroalgal group or taxa.

In order to determine which type of macroalgae is most damaging to coral, macroalgae were separated into categories by group/taxa or primary competitive mechanism. This included the commonly regarded functional groups: articulate coralline algae (ACA), CCA, *Halimeda*, turf algae and cyanobacteria [39] (table 1 and figure 2). While cyanobacteria are not taxonomically considered ‘algae’, this group of photosynthetic bacteria (colloquially referred to as blue-green algae or microalgae) are prominent competitors to corals and often included in studies of coral–algal competition (e.g. [25,38]). Calcifying macroalgae of the genus *Halimeda* were not separated by species but included a range of small- and large-segmented species common across Heron Reef, including: *Halimeda discoidea*, *Halimeda heteromorpha*, *Halimeda macroloba* and *Halimeda opuntia*, among others [34,35]. All other macroalgae were further differentiated into two groups: (i) those that compete primarily through chemical mechanisms, or (ii) those that compete primarily through physical mechanisms based on the literature (table 1). ‘Chemical’ macroalgae compete through the transfer of allelochemicals via direct contact, and are often considered the most damaging to competing coral [19,23,30]. On the other hand, macroalgae that compete primarily using physical mechanisms (e.g. abrasion) are often found to result in no visible effects (e.g. coral bleaching) to competing coral [19,23,36].

### 2.1. Statistical analyses

The outcome of coral–algal competition was explored using generalized linear mixed effect models with a binomial distribution (‘glmer’ within the *lmer4* package) [40]. Outcome was evaluated as the binary state, with neutral/coral ‘winning’ (1) assessed against coral ‘losing’ (0). We fit all possible model combinations using the predictor variables: (i) season (spring, summer, autumn, winter), (ii) algal group (ACA, CCA, chemical, cyanobacteria, *Halimeda*, physical, turf algae), and (iii) interactive effect of season and algal group (electronic supplementary material, table S1). The interaction between season and algal group was further explored by investigating the proportion of coral colonies losing (number of coral colonies losing/total number of interactions) using a linear mixed effects model. Site was included as a random effect in all models. Models were compared and the best model was selected using Akaike information criterion for small sample sizes (AICc) and AICc weight (*ω*_i_) (electronic supplementary material, table S1).

The significance of fixed effects and their interactions was determined using an analysis of variance with a type III error structure (‘Anova’ in *car* package [41]). Significant interactive effects were followed by pairwise comparison of estimate marginal means using the *emmeans* package with Tukey HSD adjusted *p-*values [42]. Data were tested for homogeneity of variance and normality of distribution through graphical analyses of residual plots for all models. All statistical analyses were done using R v. 4.0.0 software [43], and graphical representations were produced using the package *ggplot2* [44].

## 3. Results and discussion

A total of 6589 coral–algal interactions were recorded over the 2 year study at Heron Reef, representing 30 coral and 24 macroalgal taxa or groups (table 1). The majority of interactions (4031 or 61%) were recorded as coral losing, with 2279 (35%) scored neutral and only 279 (4%) of interactions identified as coral winning.

Cyanobacteria and turf algae were the most damaging to competing coral regardless of season (figure 2; electronic supplementary material, tables S2–S4). Interactions with cyanobacteria resulted in visible damage to 100% of coral colonies involved in interactions, whereas interactions with turf algae resulted in visible damage to 95.4–99.6% of colonies (electronic supplementary material, table S4). While some cyanobacteria are opportunistic and among the first to colonize recently dead coral, other cyanobacteria can actively overgrow and outcompete coral [38]—the latter of which is most consistent with our observations (figure 2). Turf algae and cyanobacteria are effectively able to damage coral through hypoxia, altering microbial communities or trapping sediments [45–48], making them among the most damaging benthic competitors [6,24,25]. Many cyanobacteria also produce toxic secondary metabolites that are used to deter herbivory and fouling, which may contribute to their competitive success [47,49,50].

Macroalgae that compete using chemical mechanisms were generally more damaging than macroalgae that compete primarily using physical mechanisms; however, these effects varied by season (*p* = 0.006, figure 2; electronic supplementary material, tables S3 and S4). Allelopathic macroalgae were significantly more damaging in winter and spring, with visible damage observed in 94.5% and 90.2% of interactions compared to only 70.8% in summer (figure 2; electronic supplementary material, table S4). Macroalgae that compete primarily through physical mechanisms displayed a non-significant shift in competitive ability, with the percentage interactions resulting in visible damage decreasing to just over half (56.2%) in summer from three-quarters (72.4–77.3%) in all other seasons (figure 2; electronic supplementary material, table S4). The increase in competitive ability in winter and spring directly coincides with the seasons in which macroalgal abundance is highest [2,11]. The increased cover not only multiplies the frequency of coral–algal contact [2], but increases macroalgal biomass per unit area, or the thickness of the macroalgal cover [11,51]. In the seasons when macroalgae are most abundant, macroalgae can cover large areas and completely surround or smother coral (figure 1), which may contribute to their competitive success. In summer, the interaction of high temperature and high light has been shown to reduce the abundance of macroalgae at Heron Reef [2] and elsewhere across the Great Barrier Reef, increasing temperatures are negatively correlated to macroalgal abundance [52]. In addition to the warmer conditions that coincide with summer leading to a decline in macroalgal cover, these conditions may also reduce (i) the production of chemical defences [53] and/or (ii) physical toughness [54], ultimately decreasing the competitive ability of both chemically rich and physically tough macroalgae.

Previous studies that have not considered seasonal shifts in competitive ability have also found chemically rich macroalgae to be more damaging than macroalgae that are physically tough [17,23,30]. The effects of physically competitive macroalgae, however, are dependent on macroalgal density, duration of contact and thalli structure [19,51,55]. The group of macroalgae defined as ‘physical’ in this study included a range of genera (greater than 10; table 1) representing diverse thalli structures from mat-forming (e.g. *Colpomenia*) to clumped and rubbery (e.g. *Hypnea*) to erect and abrasive (e.g. *Turbinaria*). A previous study that compared mat-forming and canopy-forming macroalgae on coral found that mat-forming algae (e.g. *Hydroclathrus*) significantly reduce light (by 96%) and dissolved oxygen (by 26%), with thicker algal mats resulting in the greatest mortality [51]. Although canopy-forming macroalgae (e.g. *Sargassum*) also significantly decreased light availability, these algae allowed for greater water exchange, resulting in minimal effects to understory corals [51]. Macroalgae that grow in flat dense mats (e.g. *Colpomenia*, *Hydroclathrus*) or clumps of rubbery branches (e.g. *Hypnea*, *Chnoospora*) are often seasonal, lasting only a few weeks in spring [2,10,11]. However, these ephemeral macroalgae may result in the greatest physical damage to competing corals owing to their ability to cover large areas and completely surround or smother coral [11,51] (figure 1). While perennial species often receive the most attention, future studies should incorporate ephemeral types of macroalgae as they may play an elusive, yet important role in coral reef ecosystem degradation.

Coral colonies were the most successful against ACA, *Halimeda* and CCA. In summer, a greater proportion of corals were losing against CCA when compared with all other seasons (*p* = 0.006) (figure 2; electronic supplementary material, tables S3 and S4). At Heron Reef, the abundant CCA species *Porolithon onkodes* was found to display maximum vertical growth and calcification in spring, with Lewis *et al*. [56] suggesting that *P. onkodes* may direct resources towards competition as opposed to growth in summer. The reasoning of Lewis *et al*. [56] is supported by the results presented in this study, where contact with CCA resulted in visible damage to competing coral in approximately 30% of interactions in summer, compared to only 0–5% in all other seasons (figure 2; electronic supplementary material, table S4). ACA and *Halimeda* displayed no seasonal shifts in competitive ability (figure 2; electronic supplementary material, tables S3 and S4), with a previous study also finding no temporal changes in the competitive ability of *H. heteromorpha* [36]. ACA was more damaging than *Halimeda*, with 33.5–43.9% of coral colonies losing interactions with ACA when compared with 3.2–14.5% for *Halimeda* (figure 1; electronic supplementary material, tables S3 and S4). While both ACA and *Halimeda* are articulate and calcareous, other types of macroalgae were often observed growing on ACA, potentially creating a harmful multi-species assemblage. *Halimeda* has been found to be less damaging than other macroalgal groups in a number of previous studies from the Indo-Pacific [25,46,57]. In the Caribbean, however, *Porites* and *Agaricia* were found to bleach in 90–95% of interactions with *H. opuntia* [21]. Although *Halimeda* is characterized as chemically rich [19,21,58], competition with coral has been shown to compromise the chemical defences of *Halimeda* [21]. Furthermore, older, strongly calcified portions are generally less chemically active than new, less calcified segments [58]. Chemical defences decrease quickly, only 48 h after production; therefore, exposure to the most harmful allelochemicals may be limited [58]. Although no visible damage was observed, persistent (e.g. greater than two months) contact with *Halimeda* has been shown to lead to reductions in calcification rates of competing coral [36]. Similarly, Tanner [16] found that by removing competing algal assemblages including *Halimeda*, coral did better, resulting in increased coral cover and a twofold increase in fecundity. The results of Tanner [16] and Brown *et al*. [36] highlight the complexity of these interactions, and reinforces that the methodology used in this study is limited in its resolution. The effects of coral–algal contact go beyond visible effects and should be the focus of future studies.

## 4. Conclusion

Seasonality has long been considered an important driver of macroalgal biomass and composition [11,12]. Most coral reef research, however, has not incorporated seasonal variability adequately into ecological assessments and experiments [59]. By investigating a reef system comparatively unexposed to anthropogenic impacts, we demonstrate here that macroalgae has the ability to alter its competitive ability in seasonal cycles that can be related to abundance. It remains to be determined whether seasonal shifts in biomass, the production of chemical defences and/or physical toughness are responsible for the patterns we observed. If we are to comprehensively understand coral–algal competition in natural yet changing settings, future studies could explore seasonal variability in anthropogenically disturbed reef environments, with a focus on the long-term fate of these types of interactions. Nevertheless, this study contributes to our understanding of the complicated dynamics influencing the outcomes of coral–algal interactions, which have important implications for understanding the trajectory and resilience of coral reef ecosystems into the future.

## Supplementary Material

Supplementary Information

Reviewer comments

## Data accessibility

The datasets and scripts generated/analysed for this study can be found as an electronic notebook on https://github.com/imkristenbrown/RSOS_2020.
