# Supplementary material for: Seasonal shifts in the competitive ability of macroalgae influence the outcomes of coral–algal competition
Source: R Soc Open Sci. 2020 Dec 23;7(12):201797. doi: 10.1098/rsos.201797 (PMC7813255; doi:10.1098/rsos.201797)
Supplement: Supplementary Information [file rsos201797supp1.docx]

Supplementary Information for:

**Seasonal shifts in the competitive ability of macroalgae influence the outcomes of coral-algal competition**

K. T. Brown, D. Bender-Champ, O. Hoegh-Guldberg and S. Dove

Supplementary figure


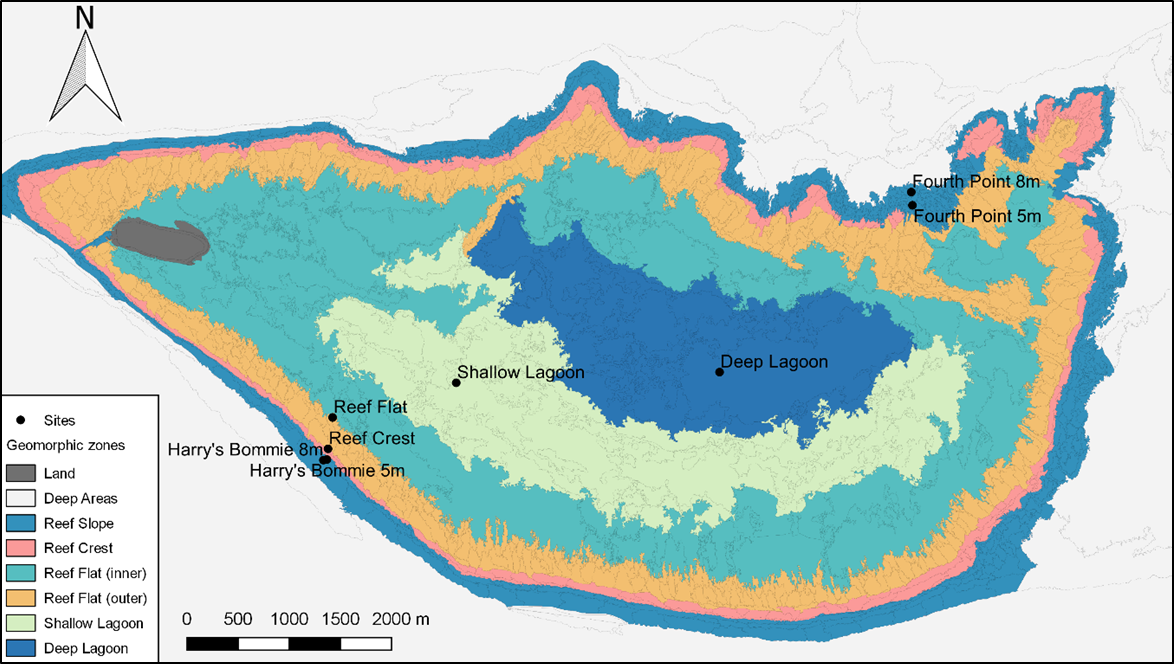


Figure S1. Geomorphological habitats of Heron Reef, southern Great Barrier Reef. Surveyed sites are indicated by point and name. Map data provided by Phinn et al. (2012).

Supplementary tables

Table S1. Generalized linear mixed model (GLMER) fits for all models examining ecological parameters influencing the outcome of coral-algal competition. Shown are the model structures with the predictor metrics included, degrees of freedom (df), Akaike's Information Criterion corrected (AICc), and AICc weight values (ωi). Models are sorted by lowest AICc, with the best model displayed first. All models were fit using the package lme4 with a binomial distribution.

| **Model** | **df** | **AICc** | **ωi** |
| --- | --- | --- | --- |
| glmer(Outcome~Season*Algal group+(1\|Site)) | 29 | 3504.55 | 1 |
| glmer(Outcome~Season+Algal group+(1\|Site)) | 11 | 3577.03 | 0 |
| glmer(Outcome~Algal group+(1\|Site)) | 8 | 3643.02 | 0 |
| glmer(Outcome~Algal group+(1\|Site)) | 5 | 6923.24 | 0 |

Table S2. Results of the best fit model on ecological parameters influencing the outcome of coral-algal competition.

|  | χ^2^ | df | p value |
| --- | --- | --- | --- |
| Intercept | 1.636 | 1 | 0.201 |
| Season | 9.528 | 3 | 0.023 |
| Algal group | 527.364 | 6 | <2.2e-16 |
| Season: Algal group | 95.166 | 18 | 0.000 |

Table S3. Results of linear mixed effects model examining the percentage of coral colonies losing by season and algal group.

|  | χ^2^ | df | p value |
| --- | --- | --- | --- |
| Intercept | 52.067 | 1 | 0.000 |
| Season | 0.995 | 3 | 0.802 |
| Algal group | 363.377 | 6 | <2.2e-16 |
| Season: Algal group | 36.428 | 18 | 0.006 |

Table S4. Mean, standard deviation (SD), standard error (SE), and confidence interval (CI) values of the percentage of coral colonies losing by algal group and season (n=3), sorted from most damaging to least.

| Algal group | Season | Percentage of coral colonies losing | SD | SE | CI |
| --- | --- | --- | --- | --- | --- |
| cyanobacteria | Autumn | 100.00% | 0 | 0 | 0 |
| cyanobacteria | Spring | 100.00% | 0 | 0 | 0 |
| cyanobacteria | Summer | 100.00% | 0 | 0 | 0 |
| cyanobacteria | Winter | 100.00% | 0 | 0 | 0 |
| turf algae | Autumn | 99.56% | 0.02156 | 0.00364 | 0.00741 |
| turf algae | Winter | 99.34% | 0.03336 | 0.00581 | 0.01183 |
| turf algae | Summer | 97.06% | 0.16667 | 0.02778 | 0.05639 |
| turf algae | Spring | 95.36% | 0.17815 | 0.03011 | 0.0612 |
| allelopathic | Winter | 94.50% | 0.12512 | 0.01886 | 0.03804 |
| allelopathic | Spring | 90.20% | 0.2208 | 0.03582 | 0.07258 |
| allelopathic | Autumn | 80.34% | 0.32917 | 0.04962 | 0.10008 |
| physical | Autumn | 77.33% | 0.28074 | 0.06126 | 0.12779 |
| physical | Spring | 73.80% | 0.32374 | 0.06118 | 0.12553 |
| physical | Winter | 72.44% | 0.37179 | 0.07155 | 0.14707 |
| allelopathic | Summer | 70.82% | 0.32952 | 0.05651 | 0.11498 |
| physical | Summer | 56.15% | 0.46552 | 0.10158 | 0.2119 |
| ACA | Spring | 43.87% | 0.4882 | 0.14093 | 0.31019 |
| ACA | Autumn | 39.58% | 0.46398 | 0.0928 | 0.19152 |
| ACA | Summer | 39.39% | 0.49031 | 0.14783 | 0.32939 |
| ACA | Winter | 33.52% | 0.41039 | 0.10596 | 0.22727 |
| CCA | Summer | 28.85% | 0.4343 | 0.11213 | 0.2405 |
| *Halimeda* | Winter | 14.51% | 0.20915 | 0.04361 | 0.09044 |
| *Halimeda* | Spring | 9.91% | 0.27619 | 0.05524 | 0.114 |
| *Halimeda* | Autumn | 8.25% | 0.19598 | 0.0352 | 0.07189 |
| CCA | Autumn | 5.56% | 0.21183 | 0.04077 | 0.0838 |
| CCA | Winter | 3.21% | 0.09307 | 0.02257 | 0.04785 |
| *Halimeda* | Summer | 3.18% | 0.07653 | 0.01446 | 0.02967 |
| CCA | Spring | 0.00% | 0 | 0 | 0 |
