# Supplementary material for: Seasonal shifts in the competitive ability of macroalgae influence the outcomes of coral–algal competition
Source: R Soc Open Sci. 2020 Dec 23;7(12):201797. doi: 10.1098/rsos.201797 (PMC7813255; doi:10.1098/rsos.201797)
Supplement: Reviewer comments [file rsos201797_review_history.pdf]

THE ROYAL SOCIETY
PUBLISHING

ROYAL SOCIETY
OPEN SCIENCE

Seasonal shifts in the competitive ability of macroalgae influence the outcomes of coral–algal competition

Kristen T. Brown, Dorothea Bender-Champ, Ove Hoegh-Guldberg and Sophie Dove

Article citation details
R. Soc. open sci. **7**: 201797.
http://dx.doi.org/10.1098/rsos.201797

Review timeline
Original submission: 12 October 2020
Final acceptance: 6 November 2020

Note: Reports are unedited and appear as submitted by the referee. The review history appears in chronological order.

Note: This manuscript was transferred from another Royal Society journal with peer review.

Review History

Decision letter (RSOS-201797.R0)

We hope you are keeping well at this difficult and unusual time. We continue to value your support of the journal in these challenging circumstances. If Royal Society Open Science can assist you at all, please don't hesitate to let us know at the email address below.

Dear Ms Brown:

It is a pleasure to accept your manuscript entitled "Seasonal shifts in the competitive ability of macroalgae influence the outcomes of coral-algal competition" in its current form for publication in Royal Society Open Science. The comments of the reviewer(s) who reviewed your manuscript are included at the foot of this letter.

Please ensure that you send to the editorial office an editable version of your accepted manuscript, and individual files for each figure and table included in your manuscript. You can send these in a zip folder if more convenient. Failure to provide these files may delay the processing of your proof. You may disregard this request if you have already provided these files to the editorial office.

You can expect to receive a proof of your article in the near future. Please contact the editorial office (openscience_proofs@royalsociety.org) and the production office (openscience@royalsociety.org) to let us know if you are likely to be away from e-mail contact -- if

Reports © 2020 The Reviewers; Decision Letters © 2020 The Reviewers and Editors; Responses © 2020 The Reviewers, Editors and Authors. Published by the Royal Society under the terms of the Creative Commons Attribution License http://creativecommons.org/licenses/by/4.0/, which permits unrestricted use, provided the original author and source are credited

you are going to be away, please nominate a co-author (if available) to manage the proofing process, and ensure they are copied into your email to the journal.

Due to rapid publication and an extremely tight schedule, if comments are not received, your paper may experience a delay in publication.

Please see the Royal Society Publishing guidance on how you may share your accepted author manuscript at https://royalsociety.org/journals/ethics-policies/media-embargo/.

Thank you for your fine contribution. On behalf of the Editors of Royal Society Open Science, we look forward to your continued contributions to the Journal.

Kind regards,
Anita Kristiansen
Editorial Coordinator

Royal Society Open Science
openscience@royalsociety.org

on behalf of Dr Melita Samoilys (Associate Editor) and Professor Pete Smith (Subject Editor)

Follow Royal Society Publishing on Twitter: @RSocPublishing
Follow Royal Society Publishing on Facebook:
https://www.facebook.com/RoyalSocietyPublishing.FanPage/
Read Royal Society Publishing's blog:
https://royalsociety.org/blog/blogsearchpage/?category=Publishing
